# Impact of Mobile Phase Composition on Separation Selectivity of Labeled Dextran Ladder in Hydrophilic Interaction Liquid Chromatography

**DOI:** 10.3390/molecules30061327

**Published:** 2025-03-15

**Authors:** Matjaž Grčman, Niko R. Pompe, Drago Kočar, Matevž Pompe

**Affiliations:** Faculty of Chemistry and Chemical Technology, University of Ljubljana, 1000 Ljubljana, Slovenia; matjaz.grcman@fkkt.uni-lj.si (M.G.); drago.kocar@fkkt.uni-lj.si (D.K.)

**Keywords:** HILIC, dextran ladder, FLD, ionic strength, organic modifier

## Abstract

The glycosylation process plays a crucial role in the structural integrity and biological activity of glycoproteins, where glycans are attached to a protein backbone. There are many kinds of glycans, the most common being N-glycans, which can be arranged into three classes, that is, complex, hybrid, and high mannoses, forming a structurally very diverse set of polar compounds that are difficult to detect and separate. Most commonly, N-glycans are labeled before separation by charged or fluorescence tags for better MS or fluorescence detection, respectively. This study examines the influence of ionic strength and organic modifier selection on the separation of fluorescently labeled dextran ladders in Hydrophilic Interaction Liquid Chromatography (HILIC). Using a Glycan BEH Amide column and varying the ammonium formate buffer concentration along with acetonitrile and methanol ratios, we investigated analyte retention, separation efficiency, and post-column conductivity changes. Our findings reveal that changes in the ionic strength of the mobile phase do not contribute to changes in selectivity, neither when acetonitrile nor methanol were used as organic modifiers to the mobile phase. However, the addition of methanol significantly changes the separation mechanism where two different prevailing separations mechanisms can be identified. It was assumed that the addition of methanol influences the folding pattern of dextrans around the permanent positive charge on the added tag, which influences the changes of separation selectivity. This work presents a systematic approach to altering mobile phase composition (buffer concentration, organic modifier type) to control retention and selectivity in complex glycan analysis. The discovery that methanol significantly alters separation behavior provides a potential new method for refining HILIC separations of polar compounds.

## 1. Introduction

Hydrophilic Interaction Liquid Chromatography (HILIC) is a powerful analytical technique for efficient separation of polar compounds, outperforming reversed-phase chromatography (RP-HPLC) for polar analytes [1,2], like glycopeptides [3], sugars [4,5,6], and glycans [7,8]. The retention mechanism in HILIC is significantly more complex than RP-HPLC or normal phase chromatography, as it primarily involves the partitioning of analytes into a water-rich layer on the stationary phase, supplemented by secondary electrostatic, dipole–dipole, and hydrogen-bonding interactions [9,10,11,12]. These interactions can exhibit both attractive as well as repulsive characteristics [13], therefore HILIC retention models often deviate from a perfect linear correlation [14]. Despite the complex separation mechanism, HILIC separation has attracted huge popularity among researchers and industrial society because HILIC chromatography uses similar mobile phases to reverse phase separation, eliminating the necessity of using two separate chromatographic systems. At the same time, it is easy to achieve compatibility with MS detection. The popularity of HILIC separation leads to the development of new stationary phases, which potentially offers new retention properties and therefore new selectivity for the separation of polar organic compounds. However, to fully exploit the advantage of HILIC separation, a better understanding of the separation mechanism is needed to make the optimization of separation conditions easier.

In HILIC separation, we are mostly talking about mix mode separation since there are always several separation mechanisms present. The main separation mechanism consists of partitioning of polar organic analytes between the bulk phase and stationary water diffusion phase. The bulk layer in HILIC consists of a less polar, organic-rich environment, where analytes can migrate relatively fast, facilitating significant mass transfer between the bulk layer and stationary water layer next to the stationary phase [6,12,13]. Due to the polar nature of the stationary phase, a water diffusion layer forms between the stationary phase and the bulk layer [15,16,17]. This diffusion layer acts as a transitional boundary, allowing selective interaction of polar analytes with the stationary phase while maintaining the less polar bulk layer environment outside. This layered configuration enables selective control over analyte retention and separation based on their polarity and affinity towards the polar stationary phase [9]. Besides the mentioned retention mechanisms, HILIC separation columns can possess additional retention mechanisms, that is, electrostatic, dipole–dipole, and the formation of hydrogen bonds. It is useful to select HILIC separation columns with a limited number of separation mechanisms in order to make observation and consequent modelling easier. Usually, column manufacturers provide information about the dominant separation mechanisms. One such column that is enabling mainly partitioning with weak ionic exchange capabilities is the BEH column.

The modern UHPLC columns provide significant advantages in Hydrophilic Interaction Liquid Chromatography (HILIC), especially under challenging conditions involving high water content or methanol as the organic phase and therefore high working pressure. One example of such columns is the BEH column, which is known for its improved stability in aqueous and basic environments compared to traditional silica-based columns, which are prone to rapid degradation under similar conditions [1]. The ethylene-bridged hybrid (BEH) particles in these columns reduce hydrolytic degradation due to their robust particle structure and high surface coverage of amide groups [18]. This composition not only prolongs column life but also enhances separation efficiency for polar analytes, making it highly suitable for complex analyses such as glycan profiling, where precise retention and selectivity are critical. Consequently, the BEH Amide column enables consistent performance even under varied mobile phase conditions, contributing to reliable and high-quality separation outcomes.

Selectivity during separation is mainly obtained through appropriate selection of the stationary phase. Nevertheless, the variations of mobile phase composition can provide additional changes to selectivity that are necessary for the separation of very complex mixtures. In order to tune separation selectivity, one can change the gradient profile, organic modifier, and ionic strength of the buffer solution.

Many applications have been published in the scientific literature using a gradient elution mixture of acetonitrile and an appropriate buffer—typically ammonium formate [1,19,20]. In general, a high amount of organic modifier shifts the distribution of polar organic compounds toward the aqueous diffusion layer and therefore increases their retention. Acetonitrile does not influence just the partitioning of analytes, but through changes to the ionic strength of a particular mixture, influences possible additional polar interaction with the stationary phase as well. The application of methanol as an alternative organic modifier is quite common in classical reverse phase chromatography, but only limited studies were reported using it as organic modifier in HILIC separation [21].

On the other hand, the influence of ionic strength is well documented. The ionic strength of the mobile phase significantly influences retention of analytes on HILIC columns, as it affects the water layer formed on the polar stationary phase. Previous findings indicate that increasing buffer strength enhances the water layer thickness on silica gel, affecting retention: weak acids retained more due to reduced repulsion, while weak bases showed shorter retention times because of increased competition from ammonium ions [7,22].

The main aim of our investigation was to study the influence of the composition of the mobile phase on the selectivity of separation in HILIC chromatography. In order to differentiate between different retention mechanisms, an experiment was designed in such a way that one can identify the contributions of the individual factors during the mix-mode separation. In our study, we investigated the separation of a fluorescently labeled dextran ladder, using the commercially available derivatization reagent RapiFluor-MS™ [23]. Dextran standards help in calibrating the retention times of real samples by providing a clear and predictable separation pattern [24]. The chemical structure of the RapiFluor-MS dextran calibration ladder is based on dextran derivatized through ethanolamine reductive amination, followed by labeling via an NHS-carbamate reaction. This process results in an ethanolamino-urea linkage that connects the sugar backbone to the fluorescent quinolinyl fluorophore and a tertiary amine group, which acts as a charge tag for MS detection. The introduction of a permanent positive charge enhances MS sensitivity but also introduces possible cation-exchange interactions with deprotonated silanol groups of the silica stationary phase. Since such modifications are commonly used in glycan and sugar analysis to improve MS response, they must be considered when interpreting retention behavior. In the described system, we tested the influence of aprotic and hydrogen bond-forming organic modifiers on retention properties, as well as variations in buffer ionic strength.

## 2. Results and Discussion

### 2.1. Impact of Organic Phase Selection on Dextran Ladder Separation

Most HILIC separation columns that are used for glycan analysis can be considered as mixed mode columns, enabling partitioning, ion-exchange, dipole–dipole interactions, and the formation of hydrogen bonds as a retention mechanism. As mentioned above, the separation column with simple separation mechanisms was selected to study the influence of mobile phase on the selectivity of separation. According to the manufacturer, the Glycan BEH Amide separation column is used predominantly for partitioning in the water diffusion layer as a retention mechanism with weak ion-exchange capabilities. The manufacturer of the dextran ladder standard, which can be used for calibrating chromatograms of real glycan samples on a Glycan BEH Amide column, recommends using ammonium formate at pH 4.4 and acetonitrile as the organic modifier [25].

**Figure 1:**
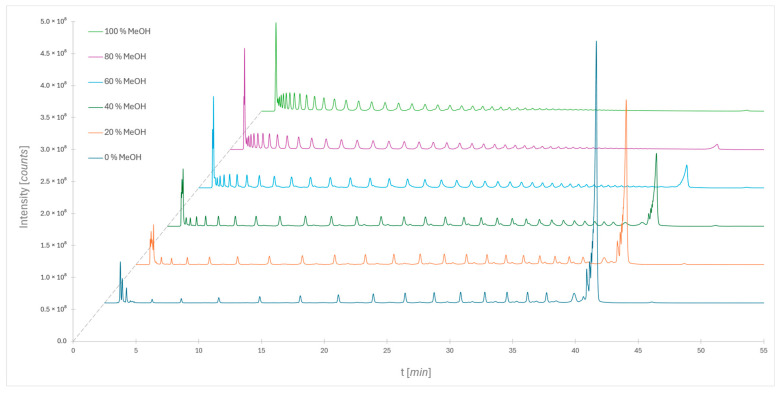
Separation of the dextran ladder using different volume ratios of methanol and acetonitrile in mobile phase B. Mobile phase A is an aqueous buffer solution (50 mM ammonium formate; pH = 4.4). A gradient separation (as shown in Table 1) was performed using a Glycan BEH Amide column.

Figure 1 (dark blue) shows the chromatogram of the standard using the recommended mobile phases and gradient (Table 1). Additionally, various compositions of mobile phase B (organic modifier) were tested, using 20%, 40%, 60%, 80%, and 100% MeOH in acetonitrile (*v*/*v*), respectively. From the chromatograms, it is evident that good separation of sugars with lower degrees of polymerization (2 ≤ DP ≤ 15) is achieved using pure acetonitrile only. Since the recommended gradient buffer content is rapidly raised from 46% to 100% in 1.5 min, dextrans with a high degree of polymerization are not separated. This can be seen at the end of the chromatogram, where a large chromatographic peak appears, representing the elution of unresolved sugars. However, the aim of this study was not to develop a quantitative separation procedure for the whole labeled dextran ladder but to observe possible changes in the selectivity of separation when methanol was added as an organic modifier. Therefore, the applied gradient was sufficient for our observation. Better separation of these compounds can be achieved by appropriately adjusting the applied gradient.

By adding methanol to the organic phase, the elution strength of the eluent is increased without changing the gradient. For example, with 20% methanol in acetonitrile, sugars with degrees of polymerization (2 ≤ DP ≤ 21) can be completely separated. If the methanol content is further increased to 40%, sugars with degrees of polymerization (2 ≤ DP ≤ 30) can be separated. Even at higher methanol concentrations, the separation shifts further: sugars with the lowest degrees of polymerization elute in the dead volume, but it enables the separation of sugars with higher degrees of polymerization. Based on peak counting and a minimum signal-to-noise ratio (S/N) of 5, we observe that sugars with degrees of polymerization exceeding DP = 50 are still detectable. The noise level was manually assessed in the range between 32 and 35 min to ensure accurate detection limits. It is also noticeable that as the methanol content increases, the peak at the end of the chromatogram diminishes and almost disappears at 100% methanol. This indicates that sugars with the highest degrees of polymerization are all separated, while those with lower DP elute in the dead volume or are eluted before the mentioned rising buffer concentration reaches 100% (Figure 1).

### 2.2. Impact of Ionic Strength on Dextran Ladder Separation

**Figure 2:**
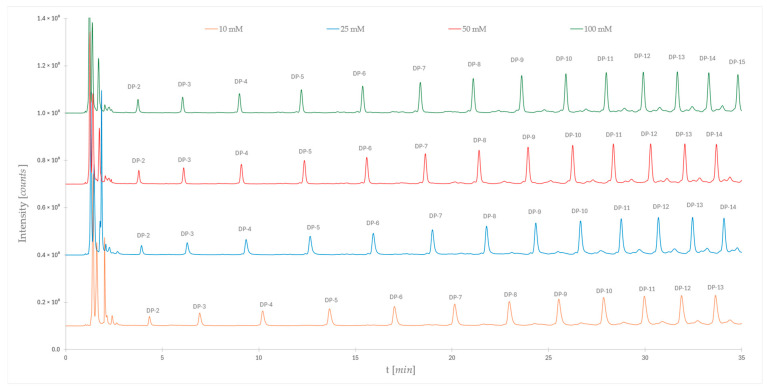
Representation of the separation of the dextran ladder using different concentrations of aqueous buffer solutions (ammonium formate; pH = 4.4) as mobile phase A. Mobile phase B is pure acetonitrile. Gradient separation, see Table 1.

The effect of different ionic strengths of the mobile phase on retention properties of labeled dextrans was investigated next. We used 10 mM, 25 mM, 50 mM, and 100 mM ammonium formate buffer concentrations with pH 4.4. Again, the recommended gradient in Table 1 was applied. The results are presented in Figure 2. The initial test was performed using just acetonitrile as the organic modifier. It is important to mention that the labeling of dextrans with RapiFluor_MS^TM^ introduces a permanent positive charge to the molecule, therefore, electrostatic interactions with ionized silanol groups on silica cannot be excluded besides the dominant partitioning retention mechanism.

The shift of retention times towards shorter times with an increasing concentration of buffer is observed (Figure 2). This effect is expected since the higher buffer concentration reduces electrostatic interactions, as well as reducing the solubility of analytes in the water diffusion layer, meaning that we are reducing both retention mechanisms. The changes in the retention times of individual dextrans are more pronounced with lower concentrations of buffer. Just a small shift in retention is observed when the buffer concentration is changed from 50 to 100 mM.

**Figure 3:**
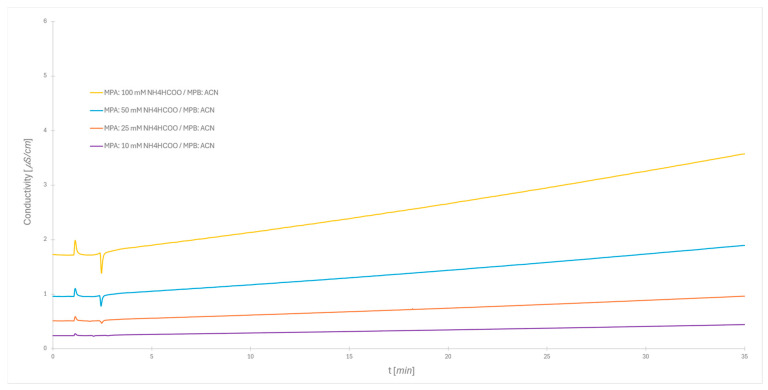
Post-column monitoring of mobile phase conductivity during Gradient 1 separation at different ionic strengths (10 mM, 25 mM, 50 mM, and 100 mM, respectively), with acetonitrile as the organic modifier; for gradient, see Table 1.

Since buffer is added just to mobile phase A, the proposed gradient creates a double gradient profile, that is, a gradient in polarity and a gradient in ionic strength. The latter can be seen from the profile of the conductivity measurements during the separation run (Figure 3). Once more, the chromatographic conditions were set according to recommendations of the standard producer. The conductivity difference between the start of the chromatogram and end of the gradient (35 min) is 0.2 μS/cm for 10 mM buffer, whereas for the 100 mM buffer, it reaches 2.0 μS/cm. When organic solvents are added, conductivity often follows a non-linear trend, rather than a simple linear relationship, because of possible ion-pairing, ion mobility changes, and the solvent dielectric constant [26,27]. The linear relationship shown in Figure 3 suggests that acetonitrile as an aprotic solvent does not form any of such interactions. This aligns with the expectations, as the change in reading follows a clear linear trend in all cases.

### 2.3. The Influence of Organic Modifier on Separation Selectivity

**Figure 4:**
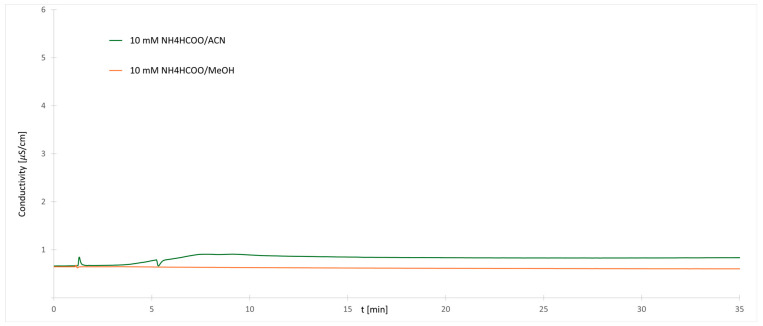
Changes of conductivity during the separation using constant ionic strength for methanol or acetonitrile as organic modifier.

To evaluate the influence of the selected organic modifier on the separation selectivity of labeled dextrans, we have changed separation gradients in such a way to enable as wide as possible separation of various dextrans, while at the same time retaining constant buffer ionic strength. Special attention was given also to the equilibration phase. The buffer solution was therefore placed in both eluents (A and B), so its concentration was constant during the whole separation process (Table 2). The results of conductivity measurements for 10 mM solution of buffer are shown in Figure 4. The results are shown for when pure acetonitrile or pure methanol was used as organic modifier.

One can see that changes in the conductivity readings are much lower than before. The changes are smaller when methanol is used as the organic phase compared to acetonitrile. The observed small changes in conductivity are not connected to changes in buffer concentration but are caused by changes in ion mobility and possible dissociation of the buffer. The acetonitrile is an aprotic solvent, and salts can be solubilized in such a solvent as ion pairs, which do not contribute to the conductivity since they are acting as neutral [26,28]. During the gradient separation process, the concentration of acetonitrile is slowly reduced and buffer salts become solubilized in water where they act as classical completely dissociated molecules, resulting in a slow increase in conductivity. The same effect is not visible in methanol mixtures.

**Figure 5:**
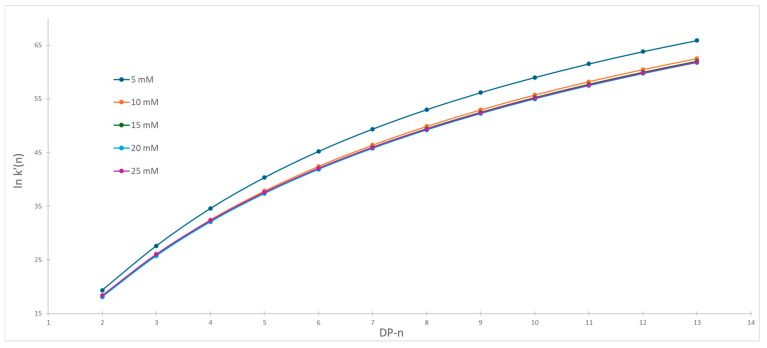
Capacity factor vs. degree of polymerization when acetonitrile was used as organic modifier.

**Figure 6:**
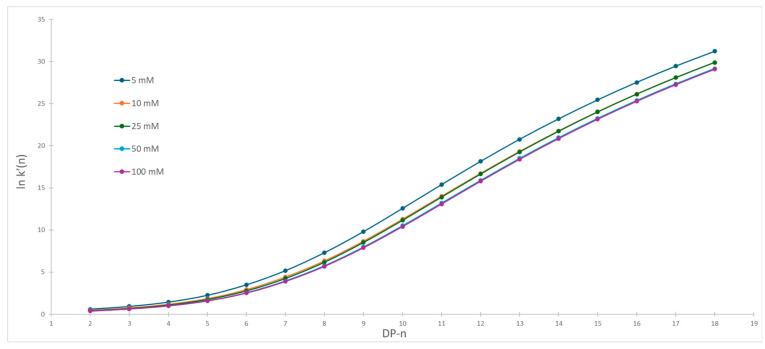
Capacity factor vs. degree of polymerization when methanol was used as organic modifier.

The newly optimized gradient conditions with fixed buffer concentration were used for the observation of the influence of various organic modifiers on separation selectivity at different buffer concentrations. For both organic modifiers, that is, methanol and acetonitrile, we measured the separation of labeled dextrans using the gradient shown in Table 2. The retention time and void time were obtained from chromatograms and capacity factors were calculated. Figure 5 and Figure 6 show the dependence of the capacity factor on the degree of polymerization at various buffer concentrations for acetonitrile and methanol, respectively.

The first observation showed that we are obtaining very similar separation patterns within the same organic modifier. On the other hand, significant change in separation patterns is observed when acetonitrile is substituted with methanol. The Pearson correlation coefficients between chromatograms within the same series were calculated and showed high correlation, with correlation coefficients being higher than 0.9999 and 0.999 in acetonitrile and methanol series, respectively. Each organic modifier was considered as a separate series and variation within series was used as an estimation of the random error. The significance of difference between series was evaluated with simple t-statistics. A slightly higher correlation is observed in acetonitrile series compared to methanol, but these differences are not significant. Such observation is not surprising because it is known that correlation with large numerical differences in the data set usually produces high correlation coefficients. From current data, one can conclude that significant changes in selectivity occur when the mobile phase is changed from acetonitrile to methanol. On the other hand, such changes are much smaller when buffer concentration is changed.

**Figure 7:**
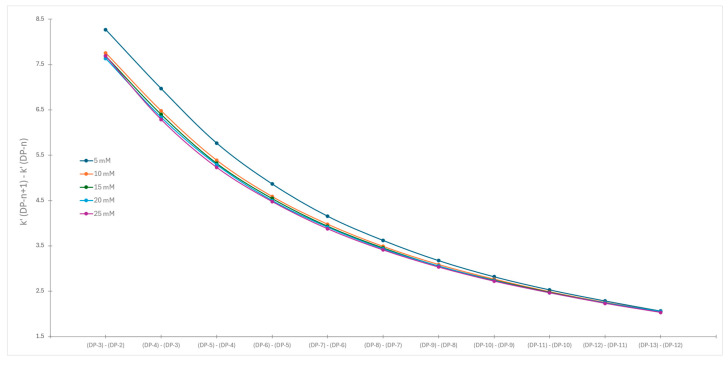
Changes in capacity factors of consecutive chromatographic peaks vs. degree of polymerization when acetonitrile was used as organic modifier.

**Figure 8:**
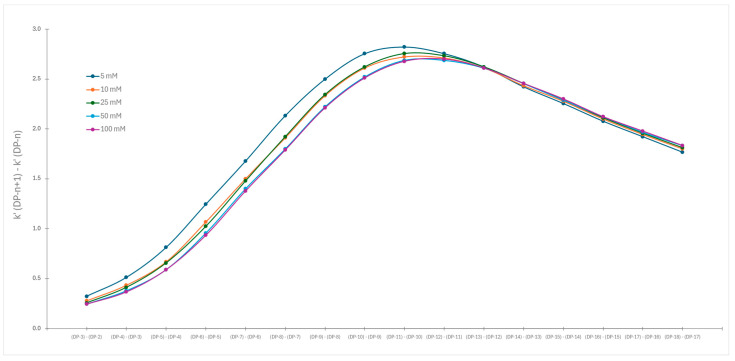
Changes in capacity factors of consecutive chromatographic peaks vs. degree of polymerization when methanol was used as organic modifier.

To emphasize the similarities and differences in the obtained datasets, the differences in capacity factors for the consecutive eluted chromatographic peaks were calculated and plotted against the degree of polymerization. Mathematically we have plotted the differential of the original retention curve vs. degree of polymerization. The described correlations using acetonitrile and methanol are presented in Figure 7 and Figure 8, respectively.

In both cases, we observe bigger differences between capacity factors for the same labeled dextran obtained for various buffer concentrations for the smaller degree of polymerization. At the end of chromatograms, the changes in capacity factors become smaller. The differentiation of original capacity factor curves reveals significant differences in the separation pattern when acetonitrile is used compared to methanol.

In the first case, one can assume the presence of the same retention mechanisms, either a single one or several, during the whole chromatographic runs. We can see a steady decline in differences of capacity factors of consecutively eluted chromatographic peaks. Such observation is caused by the linear increase of elution power of the mobile phase, as the differences in the physical properties of the eluted compounds are becoming smaller. The same pattern was observed for all buffer concentrations. Steady unidirectional changes were observed also in Figure 7, where the first derivative of changes in capacity factors vs. degree of polymerization is presented. Since the physical properties of dextrans change in a similar manner, that is, initial changes in physical properties are large and then decrease with the increased DP, the obtained results are expected.

However, application of protic organic solvent dramatically changes the separation. Initially, the differences in the capacity factors gradually increase with an increasing degree of polymerization. The function reaches its maximum when the degree of polymerization is 12. Afterwards, we see a constant decline in capacity factors regardless of the concentration of buffer. The same distribution was discovered for all applied ionic strengths. This difference in the application of both solvents is best visible when we are comparing Figure 7 and Figure 8, that is, the first derivative of changes in capacity factors vs. degree of polymerization. In the case of acetonitrile, we have a steady decline. However, when methanol was used, we observed a clear maximum at the same degree of polymerization regardless of the concentration of the buffer. From this observation, it is clear that, when methanol was used, the change in retention mechanisms is detected. This change is not caused by differences in the ionic strength of the mobile phase but rather changes in mobile phase composition, that is, the ratio between alcohol (methanol) and water [29]. It is known that methanol, like water, is capable of forming hydrogen bonds that can stabilize the structure of solvated molecules [30]. At the same time, longer dextrans are able to shield the positive charge in the macromolecules [31]. If such a process occurs in labeled dextrans, the present positive charge of the labeling functional group is shielded and inaccessible for possible electrostatic interaction. Although the exact mechanism of changes in retention properties is not known yet, we can state that application of methanol in HILIC separation is causing substantial changes in separation selectivity and that further investigation into this is justified.

## 3. Materials and Methods

### 3.1. Chemicals

The organic solvents acetonitrile (BAKER ANALYZED LC-MS reagent) and methanol (ULTRA Gradient HPLC Grade,) were purchased from J. T. Baker (Gliwice, Poland). Ammonium formate (Sigma-Aldrich, for LC-MS LiChropur™, ≥99.0%, St. Louis, MO, USA) and formic acid (Fluka, puriss p.a., ≥ 98%, Muskegon, MI, USA) were used in the preparation of the buffer solutions. Ultra-pure water with a resistivity of 18 megaohms per centimeter, produced by a Millipore Synergy^®^ UV-R purification system (MilliporeSigma, Burlington, MA, USA), was being used.

### 3.2. Standard

We used fluorescently labeled dextran from Waters (RapiFluor-MS™ Dextran calibration ladder, 50 μg/vial, P/N: 186007982, L/N: 0126132761, Waters Corp., Milford, MA USA) reconstituted in water as the standard.

### 3.3. Instrument

The analytical setup utilized was the Thermo Scientific Vanquish Flex UHPLC instrument equipped with a binary pump (Type F, VF-P10-A). The chromatographic system included an autosampler (FT VF-P10-A) and a column compartment C (VC-C10-A). Detection was performed using a fluorescence detector (UltiMate FLD 3400 RS) and a conductivity flow cell (PCM 3000). All modules of the chromatographic system were purchased from Thermo Fischer Scientific Inc., Waltham, MA, USA. The conductivity flow cell was positioned at the end of the chromatographic system, that is, after the fluorescence detector, enabling sequential detection of analytes as well as in-line measurement of the mobile phase conductivity.

For all analyses, a Waters Acquity™ Premier Glycan BEH Amide column (2.1 × 150 mm, 1.7 µm, 130 Å; P/N: 186009976, S/N: 01763208815301, Waters Corp., Milford, MA, USA) was used, installed on the chromatograph, where temperature was set to 70 °C. The sample injection volume was fixed at 1.0 µL to maintain high analytical precision and sensitivity. Due to the small injection volume and flow of 0.4 mL/min and taking into account that we used a UPLC system, no solvent effect was observed. Such low injection values are typical injection volumes for UHPLC systems, which are required in very complex glycan analysis.

The fluorescence detector was set with excitation at 265 nm, emission at 425 nm, and a sampling rate of 10 Hz. The obtained chromatographic data were processed and analyzed using Thermo Scientific Qual Browser Excalibur (Thermo Fischer Scientific Inc., Waltham, MA, USA, ver. 4.6.67.17).

### 3.4. Measurement

The chromatographic measurements were performed using an equilibrated chromatographic system. The measurements were made in triplicate. The differences in retention time were less than 0.2%.

A description of the applied gradient conditions, mobile phase compositions, and additional chromatographic parameters is provided in Table 1 and Table 2.

## 4. Conclusions

This study investigates the influence of ionic strength and solvent composition on retention properties in Hydrophilic Interaction Liquid Chromatography (HILIC), aiming to provide new perspectives on analyte separation selectivity. By systematically exploring variations in conductivity with adjustments in ammonium formate concentration and organic solvent ratios—particularly with acetonitrile and methanol—this work provides insights critical for optimizing HILIC conditions for polar compounds. The aim of this work was to detect critical parameters in mobile phase composition that influence selectivity during the separation of tagged dextrans.

The chromatographic measurements were performed using an equilibrated chromatographic system in triplicate, with changes in retention time of less than 0.2%. The initial gradient was selected based on recommendations from the standard mixture producer. The ratio of acetonitrile vs. methanol was changed from 100% acetonitrile to 0%. A higher concentration of methanol increases elution strength during separation, enabling separation of highly polymerized dextrans. On the other hand, smaller molecules become coeluted.

In the second stage, the concentration of the buffer in mobile phase B was varied from 10 mM up to 100 mM concentration with the pH value set at 4.4. The higher buffer concentrations decreased retention during the separation. The results are expected since higher buffer concentrations reduce the solubility of analytes in the diffusion layer next to the stationary phase and weaken electrostatic interactions at the same time. The variations in buffer concentration do not significantly alter the selectivity of separated labeled dextrans.

The major change in selectivity was observed when acetonitrile was exchanged with methanol. In that study, a modified elution gradient was used that enables quantitative separation of smaller dextrans, which are coeluted in the previous gradient due to the higher elution power of methanol compared to acetonitrile. Even the first look at the distribution of capacity factors according to the degree of condensation of dextrans when using methanol or acetonitrile shows significant differences. These differences are even more emphasized when observing the first derivative of the aforementioned curve with respect to the degree of condensation.

On the one hand, when using acetonitrile, we observe a continuous decrease in differences of capacity factors of consecutively eluted dextrans. The differences in the physical properties of increasingly larger condensates slowly decrease, which is the reason for such observations. Under the described conditions, no significant structural changes occur in the series of labeled dextrans.

The use of methanol completely changes the story. The differences between the capacity factors increase at the beginning of the distribution and reach a maximum at the condensation of 12 glucose units, and then begin to decrease linearly. Such behavior cannot be explained by the use of only one separation mechanism. Most likely, either a switch between the two mechanisms occurs or one of the two is excluded. Interestingly, the switch occurs at the same degree of polymerization regardless of the ionic strength of the mobile phase. However, additional studies have shown that the switch depends on the amount of methanol present. The observation is most probably caused by the folding of highly condensed glucose units where structural changes slowly shield the present permanent positive charge of labeled dextrans.

The described observations represent a new perspective on the separation of polar compounds on a HILIC separation column and bring a new functional approach to the method of changing the selectivity in this type of separation. Of course, the research does not solve the entire indicated problem, for which a series of additional studies will be required.

## Figures and Tables

**Table 1:** Gradient 1. Mobile phase A: ammonium formate buffer with different concentrations; pH = 4.4, Mobile phase B: organic solvent (acetonitrile, methanol, or mixture of both).

Time [min]	Flow [mL/min]	Mobile Phase A [%]	Mobile Phase B [%]
0	0.4	25	75
35	0.4	46	54
36.5	0.2	100	0
39.5	0.2	100	0
43.1	0.2	75	25
47.6	0.4	75	25
55	0.4	75	25

**Table 2:** Gradient 2. Mobile phase A: ammonium formate buffer with different concentrations; pH = 4.4. Mobile phase B: 90% *v*/*v* organic solvent (acetonitrile or methanol). 10% *v*/*v* buffer with 10× higher concentration in comparison with mobile phase A. Such composition of solvents ensures constant ion strength through the gradient.

Time [min]	Flow [mL/min]	Mobile Phase A [%]	Mobile Phase B [%]
0	0.4	0	100
60	0.4	40	60
61.5	0.2	100	0
64.5	0.2	100	0
68.1	0.2	0	100
72.6	0.4	0	100
80	0.4	0	100

## Data Availability

The original contributions presented in the study are included in the article, further inquiries can be directed to the corresponding author.
